# *lumpGEM*: Systematic generation of subnetworks and elementally balanced lumped reactions for the biosynthesis of target metabolites

**DOI:** 10.1371/journal.pcbi.1005513

**Published:** 2017-07-20

**Authors:** Meric Ataman, Vassily Hatzimanikatis

**Affiliations:** Laboratory of Computational Systems Biotechnology, Ecole Polytechnique Federale de Lausanne (EPFL), Lausanne, Switzerland; University of Tokyo, JAPAN

## Abstract

In the post-genomic era, Genome-scale metabolic networks (GEMs) have emerged as invaluable tools to understand metabolic capabilities of organisms. Different parts of these metabolic networks are defined as subsystems/pathways, which are sets of *functional roles* to implement a specific biological process or structural complex, such as glycolysis and TCA cycle. Subsystem/pathway definition is also employed to delineate the biosynthetic routes that produce biomass building blocks. In databases, such as MetaCyc and SEED, these representations are composed of linear routes from precursors to target biomass building blocks. However, this approach cannot capture the nested, complex nature of GEMs. Here we implemented an algorithm, lumpGEM, which generates biosynthetic subnetworks composed of reactions that can synthesize a target metabolite from a set of defined *core* precursor metabolites. lumpGEM captures balanced subnetworks, which account for the fate of all metabolites along the synthesis routes, thus encapsulating reactions from various subsystems/pathways to balance these metabolites in the metabolic network. Moreover, lumpGEM collapses these subnetworks into elementally balanced lumped reactions that specify the cost of all precursor metabolites and cofactors. It also generates alternative subnetworks and lumped reactions for the same metabolite, accounting for the flexibility of organisms. lumpGEM is applicable to any GEM and any target metabolite defined in the network. Lumped reactions generated by lumpGEM can be also used to generate properly balanced reduced core metabolic models.

## Author summary

Stoichiometric models have been used in the area of metabolic engineering and systems biology for many decades. The early examples of these models include simplified *ad hoc* built metabolic pathways, and biomass compositions. The development of genome scale models (GEMs) brought a standard to metabolic network modeling. However, the vast amount of detailed biochemistry in GEMs makes it necessary to develop methods to manage the complexity in them. In this study, we developed lumpGEM, a tool that can systematically identify subnetworks from metabolic networks that can perform certain tasks, such as biosynthesis of a biomass building block and any other target metabolite. By generating alternative subnetworks, lumpGEM also accounts for the redundancy in metabolic networks. We applied lumpGEM on latest *E*. *coli* GEM iJO1366 and identified subnetworks/lumped reactions for every biomass building block defined in its biomass formulation. We also compared the results from lumpGEM with existing knowledge in the literature. The lumped reactions generated by lumpGEM can be used to generate consistently reduced metabolic network models.

## Introduction

Stoichiometric models have been extensively used since 1980s [1–3] and prediction capabilities of these networks have been proven to be very useful. The size and structure of these models varied among different studies. One of the pioneering studies on *E*. *coli* through a small stoichiometric model is performed by Varma et al. [4,5] in where the authors described the model as composed of core carbon metabolism pathways namely, glycolysis, pentose phosphate pathway, TCA cycle and formation of some by-product formations accompanied by a part of the Electron Transport Chain (ETC). This stoichiometric definition is further extended by the integration of a biomass composition formulation that is provided in the classic text published by Neidhardt [6]. In this textbook, *E*. *coli* metabolism has been explained extensively, and all the components (amino acids, lipids, DNA, RNA etc.) that constitute 1gDW of cell were reported based on previous experiments [7]. Moreover, the amounts of 12 precursor metabolites from the core carbon metabolism (erythrose-4-phosphate, ribose-5-phosphate, pyruvate, alpha-ketoglutarate, phosphoenolpyruvate etc.) along with the requirement of cofactors (ATP, NADH, NADPH) and inorganic compounds (S, NH4) to synthesize these biomass building blocks (BBB) were estimated. Such representation has been used in different studies to understand the core carbon metabolism and its relation with biomass accumulation [8,9]. This information for *E*. *coli* allowed the authors to develop a model that can describe the growth requirements and limitations of the organism without including the complex biosynthesis routes for each individual biomass building block. With such a small stoichiometric representation of the core metabolism (~50 reactions), authors were able to predict many aspects of *E*. *coli* physiology. Similar metabolic models with a reduced representation of the biosynthesis of the building blocks have been used in many other studies for *E*. *coli* and other organisms [10–15].

In the following years, with the development of sequencing and high-throughput technologies, the gene-protein-reaction (GPR) associations [16] have been improved and the number of sequenced genomes has sparked off [17,18]. This accumulation of knowledge eventually has led to the development of Genome scale metabolic networks (GEMs) [19,20], which encapsulate all the known biochemistry of organisms. These comprehensive representations of metabolism are accompanied by biomass formulations that account for the cell composition [21]. The contribution of each biomass building block is either determined empirically, or approximated from phylogenetically close species [22].

Many metabolic models for various organisms [20,23–27] and their strains [28,29] have been constructed and they proved to be extremely useful for many different purposes ranging from strain design for biosynthesis of industrial chemicals to drug discovery [30]. Although GEMs are widely used and have provided important insight and guidance, small and yet predictive models are still widely in use in different areas, such as metabolic flux analysis (MFA) and kinetic modelling. And while the biomass formulation of Neidhardt is still in use and has proven to be valid in the last 25 years, as Pramanik et al. [12] have shown, the changes in the biomass composition have significant effects on the internal fluxes, thus should be considered very carefully. In this respect, the extended and curated biochemistry in GEMs can be used to validate and to improve the approximations made by Neidhardt, and to extend it for any organism and for every biomass building block defined in biomass compositions of GEMs and to account for alternative synthesis routes.

Towards this, we developed lumpGEM, a mixed-integer linear programming algorithm that identifies all the alternative reaction subnetworks that should be used to produce a cellular metabolite or biomass building block from a defined set of metabolites. For each subnetwork, lumpGEM derives a lumped reaction that can capture the overall stoichiometry of the subnetwork, while preserving the elemental balance. The concept of identifying minimum number of reaction or enzymes to perform a certain function is not new, throughout the last two decades, different studies have been performed around this idea. Hatzimanikatis et al. developed a mixed-integer linear programming (MILP) formulation to determine the minimum number of regulatory structures to manipulate to optimize a bioprocess, along with possible alternatives [31]. In another pioneering study, Burgard et al. [32] have determined the minimum number of reactions that can sustain growth in *E*. *coli* contemporary GEM [19]. Later, the concept of finding all possible optimal solutions in metabolic networks has been introduced [33]. Elementary flux modes analysis (EFMA) has been another popular tool to analyze the metabolic networks in terms of minimal functional units [34] and used before to study nucleotide production [35]. The main limitation of EFMA is the requirement of generation of all the EFMs, which is computationally too costly to be applied to genome-scale networks. Due to this limitation, Figueiredo et al. [36] discussed the concept of *K-shortest* EFM, an MILP approach, and identified 10 minimal reaction sets that can produce lysine in *E*. *coli* and *C*.*glutamicum*. In lumpGEM, we follow a similar but an efficient approach compared to the previous studies and we have merged it with thermodynamics-based flux analysis (TFA) to account for bioenergetics constraints. With lumpGEM we can generate thousands of thermodynamically feasible minimal subnetworks that can produce a target metabolite from a set of selected precursor metabolites.

**Fig 1:**
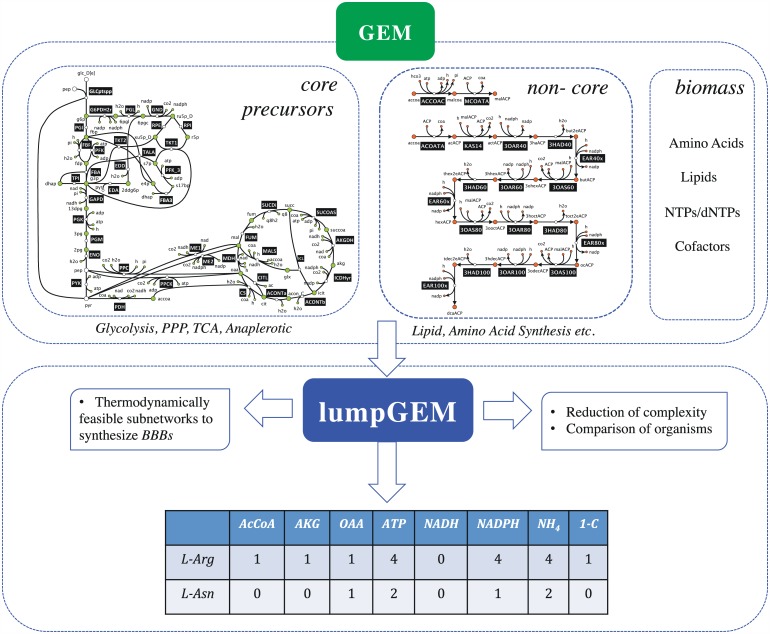
Inputs and outputs for lumpGEM. By defining the *core* precursors (AKG: alpha-keto glutarate, oxaloacetate, …), cofactor pairs (NADH, ATP, …), inorganics (SO_4_, NH_4_), biomass building blocks (*BBBs*), and *non-core* parts of metabolism, the GEM is provided to lumpGEM. The output of lumpGEM is thermodynamically feasible subnetworks, which with the *core*, is capable of synthesizing *BBBs*. The MILP characteristic of lumpGEM allows the building of alternative subnetworks and lumped reactions for the same *BBB*_*j*_, and it ranks them according to yield.

In this study, we focused on the biosynthesis pathways of biomass building blocks of *E*. *coli* iJO1366 [29], and *S*. *cerevisiae* iMM904 [37] and with lumpGEM (Fig 1), we have identified known and possible other alternative synthesis routes/subnetworks for all BBBs from *core* carbon metabolism as defined in [4,5]. We demonstrated that lumpGEM is capable of building lumped reactions in where the contribution of each *core* carbon metabolite to synthesize a biomass building block is identified and properly accounted.

Then, we performed a comparison between the values reported by Neidhardt and those generated by lumpGEM and found that lumpGEM recovered Neidhardt tables and extended them by including alternative biosynthetic routes/lumped reactions. We also performed a comparison between *E*. *coli* and *S*. *cerevisiae* for their metabolic capabilities to produce BBBs and revealed their similarities and differences. Such studies will help us to understand the capabilities of *E*. *coli* and *S*. *cerevisiae* ‘per’ biomass building block and identify the flexibility of the organism to survive by activating different parts of the metabolism to accumulate biomass. The generality of the method makes it applicable to any GEM that has a well-defined biomass composition. In addition, lumpGEM can generate lumped reactions from any part of the metabolism for any target metabolite, either a biomass building block or a biochemical and chemical compound or sets of compounds, which makes it a versatile tool to be used for different purposes.

## Results and discussion

The genome-scale models (GEMs) can simulate the growth characteristics of organism since they include the necessary biosynthetic paths to biomass building blocks (BBBs), which make up 1 gDW of cell. However, when the GEM is optimized for maximum specific growth using Flux Balance Analysis, the contribution of *M*^*core*^ (the core metabolites, for definitions, see Material and methods) to synthesize a biomass building block is not evident from the flux distribution due to the degrees of freedom that the system has, and the alternative routes that a BBB can be synthesized. In order to overcome this limitation, lumpGEM utilizes a Mixed-Integer Linear Programming (MILP) formulation (See Material and methods) to reveal the contribution of *M*^*core*^ and *R*^*core*^ (core reactions) while preserving the elemental balance. MILP formulations have been often used on biochemical networks for many purposes [31,36,38,39] since they allow the control of reactions with an on/off manner. We made use of this binary decision in order to control the flux through the reactions of *R*^*ncGEM*^ (the reactions defined in GEM network other than *R*^*core*^, (also see Postulate 1, Material and methods), and lumpGEM allowed us to build minimal subnetworks that can synthesize BBBs from any selected part of the metabolism in GEMs, in this specific case, the core carbon metabolism.

The biomass formulation defined in *E*. *coli* IOJ1366 is very well characterized and detailed and contains 102 biomass building blocks. It is mainly composed of amino acids, lipids, nucleoside triphosphates (NTPs), deoxy-NTPs and inorganic compounds (Nickel, Zinc, Iron, etc.) along with cofactors such as NAD^+^/NADH, NADP^+^/NADPH, CoA/AcCoA, and FAD. Experimental estimates of the growth associated ATP maintenance and production of diphosphate are also included in the biomass composition.

**Fig 2:**
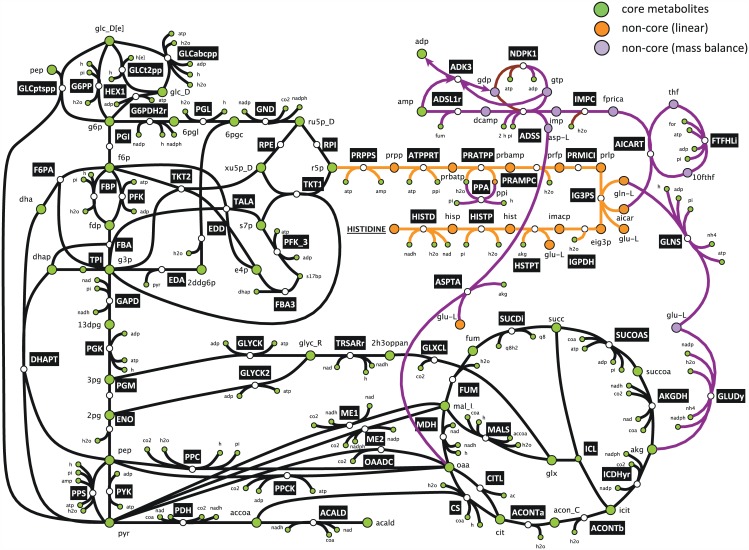
Synthesis of histidine from ribose-5-phosphate. The orange reactions are part of the linear synthesis route in the databases. The orange and purple metabolites are the *non-core* metabolites in the subnetwork. Purple reactions are balancing the non-core metabolites along the linear synthesis route. The subnetwork cannot produce any histidine from *core* network without including the purple reactions due to mass balance constraints. ‘non-core linear’ represents non-core metabolites along the linear synthesis pathway, ‘non-core mass balance’ represents the metabolite that appears as non-core within the purple reactions.

The main difference between our approach for generating synthesis routes for the BBBs and the database-based analysis is that the *subnetworks* that our method generates may include branches from linear synthesis pathways. The difference emerges from the mass conservation constraint that we force during our analysis. For instance, the smallest subnetwork that lumpGEM generated for the synthesis of histidine is composed of 21 reactions and the precursors are ribose-5-phosphate (R5P) and oxaloacetate. In the databases such as EcoCyc [40], the pathway for histidine synthesis is linear and composed of 10 steps, specifying ribose-5-phosphate (R5P) as the only precursor. When we analyse the 21-reaction subnetwork (Fig 2), we see branching points in the linear route from R5P to histidine. Due to the mass balance constraint, three metabolites, 1-(5-Phosphoribosyl)-5-amino-4-imidazolecarboxamide, L-Glutamine and diphosphate cannot be balanced in a network that is composed of *core* reactions and the linear pathway from ribose-5-phophate to histidine. Hence, the generated sets of reactions are not only the linear routes from precursor metabolites to biomass building blocks, but balanced subnetworks with stoichiometrically proportional branches (Postulate 3, Materials and methods). lumpGEM captured reactions from various subsystems that are parts of histidine subnetwork, namely alanine and aspartate metabolism, anaplerotic reactions, folate metabolism, glutamate metabolism, histidine metabolism, nucleotide salvage pathway, purine and pyrimidine biosynthesis. In addition, the lumped reaction that is generated from this subnetwork (for lumping algorithm, see Materials and methods) has only *core* metabolites, biomass building blocks and by-products on both reactants and products sides. This representation is similar to Neidhardt’s definition, since he also described the stoichiometric expenditure of *core* metabolites in his estimations. Similar to our analysis, the values that Neidhardt et al. reported for the synthesis of histidine are different than the linear route that is reported in databases. However, Neidhardt reported only 1 lumped reaction for each biomass building block, and they are not overall stoichiometrically balanced (S2 Table). lumpGEM allows us to build alternative subnetworks and corresponding lumped reactions for the same *BBB*_*j*_. In this specific case, with the minimum subnetwork size $S_{min}^{j}$ being 21, lumpGEM generated 12 alternative subnetworks, and 3 unique lumped reactions. *This signifies that the overall lumped reactions of different subnetworks can be the same*. This has been observed also in a previous study that focuses on pathway generations for an industrial chemical [41].

A comparison between lumpGEM results and Neidhardt tables for the common metabolites (ATP, NADH, NADPH, etc) indicates that they are close to each other, however the overall stoichiometry that lumpGEM reports for the biosynthesis of histidine is more detailed and includes more metabolites as co-substrates and co-products (Table 1). The main reason for this discrepancy is that Neidhardt did not report elementally balanced lumped reactions for any of the biomass building blocks. For instance, oxaloacetate appears as a precursor to balance the *non-core* metabolite L-aspartate in the subnetworks that participates in adenylosuccinate synthase reaction as a co-substrate, and is not reported in Neidhardt precursors (Table 1). Therefore, values reported by lumpGEM are more comprehensive, and account for the balancing of all metabolites in the synthesis pathways, along with the small molecules, such as inorganic metabolites and protons.

**Table 1:** Lumped reactions and statistics for amino acids.

BIOMASS BUILDING BLOCK	LUMPED REACTIONS
**L-ALANINE**	2:01:01
	H + NADPH + NH4 + PYR -> ALA-L + H2O + NADP
	Neidhardt: PYR + NADPH + NH4 -> ALA-L
**L-ARGININE**	13:02:02
	ACCoA + AKG + **3 ATP** + CO2 + 4 NADPH + 4 NH4 + OAA -> AC + **3 ADP** + ARG-L + CoA + FUM + **H** + **2 H2O** + 4 NADP + **3 PI**
	ACCoA + AKG + **4 ATP** + CO2 + 4 NADPH + 4 NH4 + OAA -> AC + **4 ADP** + ARG-L + CoA + FUM + **2 H** + **H2O** + 4 NADP + **4 PI**
	Neidhardt: AKG + 4 ATP + 4 NADPH + 4 NH4 -> NADH + ARG-L
**L-ASPARAGINE**	5:02:02
	**2 ATP** + NADPH + 2 NH4 + OAA -> **2 ADP** + ASN-L + H + NADP + **2 PI**
	**ATP** + NADPH + 2 NH4 + OAA -> **ADP** + ASN-L + H2O + NADP + **PI**
	Neidhardt: 4 ATP + 2 NH4 + OAA -> ASN-L
**L-ASPARTATE**	2:01:01
	H + NADPH + NH4 + OAA -> ASP-L + H2O + NADP
	Neidhardt: NADPH + NH4 + OAA -> ASP-L
**L-CYSTEINE**	15:06:02
	3PG + ACCoA + **4 ATP** + NAD + 5 NADPH + NH4 + SO4 -> AC + **4 ADP** + CoA + CYS-L + NADH + 5 NADP + **5 PI**
	3PG + ACCoA + **3 ATP** + H + NAD + 5 NADPH + NH4 + SO4 -> AC + **3 ADP** + CoA + CYS-L + **H2O** + NADH + 5 NADP + **4 PI**
	Neidhardt: 3PG + 4 ATP + 5 NADPH + NH4 -> CYS-L
**L-GLUTAMINE**	2:02:02
	AKG + **ATP** + NADPH + 2 NH4 -> **ADP** + GLN-L + **H2O** + NADP + **PI**
	AKG + **2 ATP** + NADPH + 2 NH4 -> **2 ADP** + GLN-L + **H** + NADP + **2 PI**
	Neidhardt: AKG + NADPH + NH4 -> GLN-L
**L-GLUTAMATE**	1:01:01
	AKG + H + NADPH + NH4 -> GLU-L + H2O + NADP
	Neidhardt: AKG + NADPH + NH4 -> GLN-L
**GLYCINE**	8:02:02
	**3PG** + **H2O** + **NAD** + NH4 -> **FOR** + GLY + **3 H** + **NADH** + **PI**
	**2 ATP** + **2 H** + **3 NADPH** + NH4 + **OAA** -> **ACALD** + **2 ADP** + GLY + **3 NADP** + **2 PI**
	Neidhardt: 3PG + NADPH + NH4 -> GLY + NAD + 1-C
**L-HISTIDINE**	21:12:03
	**5 ATP** + FOR + 2 NAD + 2 NADPH + 3 NH4 + OAA + R5P -> **5 ADP** + FUM + **8 H** + HIS-L + 2 NADH + 2 NADP + **6 PI**
	**7 ATP** + FOR + **2 H2O** + 2 NAD + 2 NADPH + 3 NH4 + OAA + R5P -> **7 ADP** + FUM + **10 H** + HIS-L + 2 NADH + 2 NADP + **8 PI**
	**9 ATP** + FOR + **4 H2O** + 2 NAD + 2 NADPH + 3 NH4 + OAA + R5P -> **9 ADP** + FUM + **12 H** + HIS-L + 2 NADH + 2 NADP + **10 PI**
	Neidhardt: 6 ATP + 1-C + NADPH + 3 NH4 -> HIS-L + 3 NAD
**L-ISOLEUCINE**	12:01:01
	2 ATP + 5 H + 5 NADPH + NH4 + OAA + PYR -> 2 ADP + CO2 + 2 H2O + ILE-L + 5 NADP + 2 PI
	Neidhardt: 2 ATP + 5 NADPH + OAA + PYR + NH4 -> ILE-L
**L-LEUCINE**	10:01:01
	ACCoA + 2 H + NAD + 2 NADPH + NH4 + 2 PYR -> 2 CO2 + CoA + H2O + LEU-L + NADH + 2 NADP
	Neidhardt: ACCoA + 2NADPH + NH4 + 2 PYR -> LEU-L + NADH
**L-LYSINE**	11:01:01
	ATP + 4 H + 4 NADPH + 2 NH4 + OAA + PYR + SUCCoA -> ADP + CO2 + CoA + 2 H2O + LYS-L + 4 NADP + PI + SUCC
	Neidhardt: 2 ATP + 4 NADPH + 2 NH4 + OAA + PYR -> LYS-L
**L-METHIONINE**	25:06:02
	2 3PG + ACCoA + **5 ATP** + **2 H** + NAD + 9 NADPH + 2 NH4 + OAA + SO4 + SUCCoA -> AC + **5 ADP** + 2 CoA + GLY + **H2O** + MET-L + NADH + 9 NADP + **7 PI** + PYR + SUCC
	2 3PG + ACCoA + **4 ATP** + **3 H** + NAD + 9 NADPH + 2 NH4 + OAA + SO4 + SUCCoA -> AC + **4 ADP** + 2 CoA + GLY + **2 H2O** + MET-L + NADH + 9 NADP + **6 PI** + PYR + SUCC
	Neidhardt: 7 ATP + 8 NADPH + NH4 + 1-S + 1-C -> MET-L
	**L-PHENYLALANINE**	11:01:01
		ATP + E4P + 2 NADPH + NH4 + 2 PEP -> ADP + CO2 + H + 2 H2O + 2 NADP + PHE-L + 4 PI
Neidhardt: E4P + 2 NADPH _ NH4 + 2 PEP -> PHE-L	
**L-PROLINE**	5:01:01
	AKG + ATP + 2 H + 3 NADPH + NH4 -> ADP + 2 H2O + 3 NADP + PI + PRO-L
	Neidhardt: ATP + AKG + 3 NADPH + NH4 -> PRO-L
**L-SERINE**	4:01:01
	3PG + NAD + NADPH + NH4 -> H + NADH + NADP + PI + SER-L
	Neidhardt: 3PG + NADPH + NH4 -> NADH + SER-L
**L-THREONINE**	7:01:01
	2 ATP + 2 H + 3 NADPH + NH4 + OAA -> 2 ADP + 3 NADP + 2 PI + THR-L
	Neidhardt: 2 ATP + 3 NADPH + NH4 + OAA -> THR-L
**L-TRYPTOPHAN**	17:02:02
	**4 ATP** + E4P + NADPH + 2 NH4 + 2 PEP + R5P -> **4 ADP** + CO2 + G3P + **6 H** + **H2O** + NADP + 7 PI + TRP-L
	**3 ATP** + E4P + NADPH + 2 NH4 + 2 PEP + R5P -> **3 ADP** + CO2 + G3P + **5 H** + **2 H2O** + NADP + 6 PI + TRP-L
	Neidhardt: 5 ATP + E4P + 3 NADPH + 2 NH4 + 2PEP + R5P -> 2 NADH + TRP-L
**L-TYROSINE**	11:01:01
	ATP + E4P + NAD + 2 NADPH + NH4 + 2 PEP -> ADP + CO2 + 2 H + H2O + NADH + 2 NADP + 4 PI + TYR-L
	Neidhardt: ATP + E4P + 2 NADPH + 2 PEP -> NADH + TYR-L
**L-VALINE**	5:01:01
	3 H + 2 NADPH + NH4 + 2 PYR -> CO2 + 2 H2O + 2 NADP + VAL-L
	Neidhardt: 2 NADPH +NH4 + 2 PYR -> VAL-L

The numbers (i:j:k) represent *i*, size of subnetworks for amino acids (2:1:1), *j*, the number of alternative subnetworks (2:1:1) and k, the number of unique lumped reactions (2:1:1), respectively. The rows under the lumped reactions represent the values reported by Neidhardt. In this table the size corresponds only to smallest size subnetworks ($S_{min}^{j}$) for each BBB.

The differences between the alternative subnetworks may emerge from different reactions in the ‘linear’ pathway from main precursor to the biomass building blocks, or from the other *non-core* reactions, which are balancing the *non-core* metabolites in the linear route. These two sources of differences, and especially the latter, may result in an explosion in the number of subnetworks that can be generated for some of the biomass building block. For metabolites like amino acids, which are not so far from the core carbon metabolism, the number of alternative smallest subnetworks $S_{min}^{j}$ is relatively small (Table 1), whereas for big molecules, such as lipids, there exists hundreds of alternative routes (S1 Table). The small number of alternative subnetworks for amino acids also shows that the number of *non-core* metabolites that appeared along the linear synthesis route is small, since the main explosion in the number of subnetworks emerges from these reactions. As an example, all 12 alternative subnetworks for histidine include the linear 10 steps route from R5P to histidine and alternative subnetworks are generated by other *non-core* reactions. Moreover, there is a slight correlation between the number of alternative subnetworks and the size of the subnetworks. Most of the amino acids that have more than 2 alternative subnetworks require more than 10 steps for their biosynthesis. A big molecule, Phosphatidylglycerol (dihexadecanoyl, n-c16:0) is a lipid with a $S_{min}^{j}$ of 40 reactions, and has 127 alternative subnetworks with 16 unique lumped reactions. In the first subnetwork generated by lumpGEM, within the 40 reactions, 34 of them are part of linear synthesis route and 6 of them are balancing *non-core* metabolites. However, despite the increase in the number of subnetworks, the number of unique lumped reactions remains relatively small (S1 Table).

When we analyse the alternative lumped reactions, in some cases, we only observe a difference in the stoichiometry of the cofactors in the reactant and product side. For example, the small change between the two alternative L-arginine lumped reactions is caused from alternative reactions that are decomposing pyrophosphate. In one case, pyrophosphate is decomposed into phosphate and water by the inorganic diphosphatase enzyme and in the second subnetwork it is decomposed into ATP, phosphate and proton using ADP as co-substrate by polyphosphate kinase enzyme. Thus, the main carbon flows of the subnetworks are exactly the same, and the lumped reactions differ only in the stoichiometry of the cofactor metabolites.

### Complex biomass components and biomass associated processes

Along with the biomass building blocks such as amino acids and lipids, the biomass formulation of iJO1366 includes growth associated maintenance (GAM) and the *de novo* synthesis of adenylate pool metabolites. The Varma core network (S2 File 2) is capable of hydrolyzing the amount of ATP for GAM in the biomass (53.95mmol/gDW). However, the stoichiometric coefficients of ATP and ADP are not the same in the biomass equation, which indicates that the biomass formulation of *E*. *coli* requires synthesis of adenylate pool metabolites. In order to identify the subnetwork(s) for the synthesis of these pool metabolites, we followed the same procedure that we did for biomass building blocks, and we built a GEM with an additional reaction with the coefficients of ATP, ADP, water, phosphate and proton from GEM biomass. Then, by forcing a flux through this reaction, and by minimizing the number of *non-core* reactions (See Material and methods), we generated minimal subnetworks *S*_*min*_. The resulting networks are composed of 27 reactions with 24 alternatives, which synthesize adenylate pool metabolite ADP.

Along with the biomass building blocks, GAM and adenylate pool metabolites, the biomass equation of iJO1366 includes diphosphate as a by-product. By following the same procedure that we have followed for adenylate pool, we generated *subnetworks* that balance the diphosphate in the biomass formulation. There are 2 alternative subnetworks composed of only 1 reaction in *R*^*ncGEM*^ that can balance the diphosphate in biomass equation: Inorganic Diphosphatase (PPA) and Polyphosphate Kinase (PPKr). Both of these reactions are decomposing the diphosphate into phosphate and proton, the former with water, and the latter with ATP/ADP cofactor pair.

### Ranking alternative lumped reactions—Yield analysis

When we analysed the alternative lumped reactions for the same biomass building block, we observe different requirement of precursors, cofactors, nitrogen and sulphur sources. This behaviour is expected, and a detailed analysis could also suggest which lumped reaction is more suitable for specific studies. One of the main criteria to rank the lumped reactions is their capability to synthesize the *BBB* from the carbon source, specifically the yield of *BBB* on the carbon source. In order to calculate the yield per lumped reaction, we built a ‘mini’ core model for each of them, which is composed of *M*^*core*^—*R*^*core*,^ *V*_*BBB*_ and the lumped reactions under study. By optimizing the synthesis of the selected *BBB* and calculating the C-mole yield over the carbon source of interest, specifically glucose, we ranked the alternative lumped reactions for each *BBB*. Interestingly, different lumped reactions can produce different amounts of biomass building blocks over a wide range of yield amounts (Table 2).

**Table 2:** The lumped reactions generated for deoxynucleoside triphosphate dTTP.

BBB	LUMPED REACTIONS	YIELD
**dTTP**	6 ATP + FOR + H + 4 NADPH + 2 NH4 + OAA + Q8 + R5P -> 6 ADP + DTTP + 3 H2O + 4 NADP + 4 PI + Q8H2	0.74
	6 ATP + FOR + H + MQN8 + 4 NADPH + 2 NH4 + OAA + R5P -> 6 ADP + DTTP + 3 H2O + MQL8 + 4 NADP + 4 PI	0.73
	8 ATP + FOR + 4 NADPH + 2 NH4 + OAA + Q8 + R5P -> 8 ADP + DTTP + H + H2O + 4 NADP + 6 PI + Q8H2	0.71
	8 ATP + FOR + MQN8 + 4 NADPH + 2 NH4 + OAA + R5P -> 8 ADP + DTTP + H + H2O + MQL8 + 4 NADP + 6 PI	0.70
	6 ATP + COA + FOR + H + 3 NADPH + 2 NH4 + OAA + PYR + Q8 + R5P -> ACCOA + 6 ADP + CO2 + DTTP + 3 H2O + 3 NADP + 4 PI + Q8H2	0.66
	6 ATP + COA + FOR + H + MQN8 + 3 NADPH + 2 NH4 + OAA + PYR + R5P -> ACCOA + 6 ADP + CO2 + DTTP + 3 H2O + MQL8 + 3 NADP + 4 PI	0.66
	8 ATP + COA + FOR + 3 NADPH + 2 NH4 + OAA + PYR + Q8 + R5P -> ACCOA + 8 ADP + CO2 + DTTP + H + H2O + 3 NADP + 6 PI + Q8H2	0.64
	8 ATP + COA + FOR + MQN8 + 3 NADPH + 2 NH4 + OAA + PYR + R5P -> ACCOA + 8 ADP + CO2 + DTTP + H + H2O + MQL8 + 3 NADP + 6 PI	0.63
	6 ATP + FOR + FUM + H + 4 NADPH + 2 NH4 + OAA + R5P -> 6 ADP + DTTP + 3 H2O + 4 NADP + 4 PI + SUCC	0.59
	8 ATP + FOR + FUM + 4 NADPH + 2 NH4 + OAA + R5P -> 8 ADP + DTTP + H + H2O + 4 NADP + 6 PI + SUCC	0.57
	6 ATP + COA + FOR + FUM + H + 3 NADPH + 2 NH4 + OAA + PYR + R5P -> ACCOA + 6 ADP + CO2 + DTTP + 3 H2O + 3 NADP + 4 PI + SUCC	0.54
	8 ATP + COA + FOR + FUM + 3 NADPH + 2 NH4 + OAA + PYR + R5P -> ACCOA + 8 ADP + CO2 + DTTP + H + H2O + 3 NADP + 6 PI + SUCC	0.52

The lumped reactions are sorted based on their *carbon mole dTTP synthesis* / *carbon-mole glucose uptake* yield.

dTTP is a deoxy nucleoside triphosphates and its main precursor for all the generated subnetworks is ribose-5-phosphate (R5P) from Pentose Phosphate Pathway. Consequently, the *core* network can supply the same amount of carbon to all the generated subnetworks and corresponding lumped reactions. One explanation for the differences in yields (Table 2) is the capability of the lumped reactions to direct all the carbon from R5P to dTTP. When we analyse the 4 lumped reactions with highest yield, we see that on the product side, the only compound other than inorganics and cofactor pairs like quinone/quinol, ATP/ADP-PI is dTTP. For the lumps with second highest yield, along with the pyruvate (C3) in the reactant side, we see AcCoA (C2) and CO_2_ (C1) on the product side. In the 3^rd^ highest yield case, fumarate (C4) replaces pyruvate on the reactant side, and succinate (C4) appears on the product side as a by-product. The lowest yield producing lumped reaction has fumarate and pyruvate on the reactant side, and has AcCoA, CO_2_ and succinate on the product side of the equation. This signifies that the lumped reactions with lower yield are losing carbon through those *core* metabolites and the number of carbons in these metabolites defines the yield. However, it is not possible to generalize such a rule, since the metabolites appearing on the product side of the lumped reaction can be assimilated by the system and the capacity of the network for this re-assimilation will have a significant effect on the yield of the lumped reaction. Moreover, having a lower yield does not necessarily mean that these lumped reactions are not useful for metabolic modelling, since they can be used under sub-optimal growth conditions or under conditions when growth is not the main physiological optimality criterion or to provide flexibility under mutation. The use of these alternatives and their physiological interpretation deserves an in-depth study and lumpGEM can serve as a framework for systematic studies.

### Generating a metabolic model with lumpGEM

By generating subnetworks for GAM and diphosphate, lumpGEM took into account all the components of biomass formulation both on the product and reactant sides. By testing for yield, we have shown that all the generated lumped reactions are capable of producing their target *BBB*_*j*_. However, to produce biomass, these lumped reactions must be able carry flux under the same quasi steady state condition, in the same model with biomass as cellular objective. This requires generating a metabolic network composed of the defined *core* network, lumped reactions, the transport and sink reactions defined in GEM. We used the Varma model (S2 File 2) as core model and the GEM iJO1366 as defined in the previous sections and introduced a new mixed-integer linear programming (MILP) formulation to have a systematic way to choose the lumped reactions. We formulated the problem as the following: We generated a model with all the lumped reactions generated by lumpGEM and calculated the theoretical maximum yield for biomass. We then fixed this yield in the model, and minimized the number of active lumped reactions and include only these lumped reactions for further analysis. This network consists of 56 cytosolic enzymatic (Varma network), 144 transport reactions, 78 lumpGEM output reactions along with 64 sinks (343 in total with biomass formulation) and 153 unique metabolites. The metabolic network is capable of producing 0.951/hr specific growth rate with 10 mmol/gDWhr glucose uptake rate. This signifies that all the lumped reactions were capable of producing corresponding *BBB*_*j*_ successfully under the given condition simultaneously. The specific growth rate of GEM for the same condition is 0.997/hr, which is close to the biomass accumulation of the model generated with the output of lumpGEM. This shows that lumpGEM can be used to generate networks, which are small, but comprehensive and able to mimic the GEM behaviour.

### Analysis on compartmentalized models, test case on *S*. *cerevisiae*

lumpGEM can be applied to any GEM with a proper biomass formulation, and in order to test its applicability, we used it on a eukaryotic, compartmentalized GEM, iMM904 [37] of *S*. *cerevisiae*. This yeast is one of the mostly studied unicellular organisms along with *E*. *coli*, and has many biotechnological applications [42]; thus making it a strong candidate for modelling approaches. Applying lumpGEM on this *S*. *cerevisiae* strain revealed the contribution of different possible precursors and cofactors for each of the biomass building blocks defined in GEM, moreover it revealed alternative subnetworks and lumped reactions for the same biomass building block. This can be interpreted as building ‘Neidhardt style’ tables for *S*. *cerevisiae*.

In order to define the core for *S*. *cerevisiae*, we used the Metabolic Flux analysis (MFA) model of Christen et al. [43] and mapped the reactions of this model with the iMM904 reactions. In addition, we mapped reactions of iMM904 that are in Varma network but not in MFA model and included them as *core* reactions for *S*. *cerevisiae* network. The generated core network is composed of (S2 File 2) 88 reactions and 67 unique metabolites along 2 compartments, cytoplasm and mitochondria. Following these steps, we have applied the lumpGEM algorithm to the GEM as described in Material Methods section. The main difference between *E*. *coli* and *S*. *cerevisiae* GEMs are that *S*. *cerevisiae* GEM is compartmentalized, however this does not bring any more complexity for lumpGEM since it treats the transport reactions between compartments as it treats single enzymatic reactions. These transport reactions are part of the generated subnetworks for biomass building blocks and can participate in lumped reactions. These lumped reactions can include metabolites from different compartments, if these compartments are included in the *ad-hoc* selected network. By the nature of the lumpGEM algorithm, the lumped reactions include only core metabolites, biomass building blocks and by-products. Since the core network has 2 compartments, mitochondria and cytosol, the resulting lumped reactions can have metabolites from these compartments. The synthesis pathways of common biomass building blocks of *S*. *cerevisiae* and *E*. *coli* such as amino acids are very similar. The sizes of the networks differ mainly from the transport reactions between the compartments. Another reason for the divergence is the non-core metabolites along the linear routes to biomass building blocks, because there are different enzymes in these two organisms that are balancing these non-core metabolites. Interestingly, different subnetworks between the two organisms do not necessarily produce different lumped reactions. When the synthesis pathway is in one compartment (mainly cytosol), the lumped reactions of *E*. *coli* and *S*. *cerevisiae* are very similar (Table 3). L-phenylalanine, L-methionine, L-serine, L-cysteine and L-tyrosine are some examples of these similarities. Small differences for these overall reactions emerge from different cofactor usage. A similar behaviour is also observed for subnetworks including more than 1 compartment. The main difference between *E*. *coli* and *S*. *cerevisiae* overall reactions emerges from metabolites in different compartments, which are also different due to the energetics cost of transport reactions. As an example, the L-arginine biosynthesis in *E*. *coli* and *S*. *cerevisiae* is very similar, however the main difference emerges from the transport reactions between the compartments in the yeast. A smallest subnetwork for *E*. *coli* is composed of 13 reactions, and for *S*. *cerevisiae*, this number rises to 17. Two additional reactions for *S*. *cerevisiae* are the transport reactions between cytosol and mitochondria for the metabolites L-aspartate and ornithine. Another difference is the bicarbonate equilibration reaction (HCO3E) converting carbon dioxide to bicarbonate, which is the co-substrate for the carbamoyl-phosphate synthase reaction converting glutamine to carbamoyl phosphate. The reaction that produces carbamoyl phosphate in *E*. *coli* model uses carbon dioxide instead of bicarbonate as co-substrate. Moreover, it does not use L-glutamine as co-substrate. Therefore, the last difference in the subnetworks emerges from the synthesis of glutamine by *S*. *cerevisiae* through the usage of glutamate synthase (NADH2) and glutamine synthetase enzymes.

**Table 3:** Lumped reactions for amino acids in *S*. *cerevisiae* using the core defined in the main text and comparison with *E*. *coli* lumped reactions.

BIOMASS BUILDING BLOCK	LUMPED REACTIONS
**L-ALANINE**	2:1:1/2:1:1
	H + NADPH + NH4 + PYR -> ALA-L + H2O + NADP *
**L-ARGININE**	17:4:3/13:2:2
	8 ATP + 2 CO2 + 4 H2O + 3 NADH + 4 NH4 + 3 OAA + AKG_M + ATP_M + NADPH_M -> 8 ADP + ARG-L + FUM + 5 H + HCO3 + 3 NAD + 8 PI + ADP_M + 2 H_M + NADP_M + 2 OAA_M + PI_M
	5 ATP + 2 CO2 + H2O + 3 NADPH + 4 NH4 + 3 OAA + AKG_M + ATP_M + NADPH_M -> 5 ADP + ARG-L + FUM + 2 H + HCO3 + 3 NADP + 5 PI + ADP_M + 2 H_M + NADP_M + 2 OAA_M + PI_M
	2 AKG + 5 ATP + 2 CO2 + H2O + 3 NADPH + 4 NH4 + OAA + ATP_M + DHLAM_M + NAD_M + NADPH_M -> 5 ADP + ARG-L + FUM + 4 H + HCO3 + 3 NADP + 5 PI + ADP_M + AKG_M + H_M + LPAM_M + NADH_M + NADP_M + PI_M
**L-ASPARAGINE**	6:2:2/5:2:2
	AMP + 5 ATP + 3 H2O + NADH + 2 NH4 + OAA + PPI -> 6 ADP + ASN-L + 5 H + NAD + 6 PI
	AMP + 4 ATP + 2 H2O + NADPH + 2 NH4 + OAA + PPI -> 5 ADP + ASN-L + 4 H + NADP + 5 PI
**L-ASPARTATE**	3:1:1/2:1:1
	CO2 + NADPH + NH4 + OAA -> ASP-L + HCO3 + NADP
**L-CYSTEINE**	14:2:2/15:6:2
	3PG + ACCOA + AMP + 4 ATP + NAD + 5 NADPH + NH4 + PPI + SO4 -> AC + 5 ADP + COA + CYS-L + H + NADH + 5 NADP + 6 PI
	ACCOA + AMP + 4 ATP + 2 GLX + 2 H + 6 NADPH + NH4 + 1 PPI + SER-L + SO4 + NH4_M -> AC + 5 ADP + COA + CYS-L + 2 GLY + 2 H2O + 6 NADP + 5 PI
**L-GLUTAMINE**	3:1:1/2:2:2
	AKG + ATP + CO2 + NADPH + 2 NH4 -> ADP + GLN-L + H + HCO3 + NADP + PI
**L-GLUTAMATE**	2:1:1/1:1:1
	AKG + CO2 + NADPH + NH4 -> GLU-L + HCO3 + NADP
**GLYCINE**	3:1:1/8:1:1
	GLX + H + NADPH + NH4 -> GLY + H2O + NADP
**L-HISTIDINE**	25:18:6/21:12:3
	6 ATP + GLX + 2 H2O + MLTHF + 2 NAD + 2 NADPH + 3 NH4 + OAA + R5P + NH4_M -> 6 ADP + FUM + GLY + 10 H + HIS-L + 2 NADH + 2 NADP + 7 PI + THF
	6 ATP + GLX + 2 H2O + MLTHF + 3 NAD + 3 NADPH + 3 NH4 + OAA + R5P + NH4_M -> 6 ADP + FUM + GLY + 10 H + HIS-L + 3 NADH + 3 NADP + 7 PI + THF
	9 ATP + GLX + 5 H2O + MLTHF + 3 NH4 + OAA + R5P + NH4_M -> 9 ADP + FUM + GLY + 13 H + HIS-L + 10 PI + THF
	3PG + 6 ATP + 3 H2O + MLTHF + 4 NAD + 3 NADPH + 4 NH4 + OAA + R5P –> 6 ADP + FUM + 12 H + HIS-L + 4 NADH + 3 NADP + 8 PI + SER-L + THF
	3PG + 9 ATP + 6 H2O + MLTHF + NAD + 4 NH4 + OAA + R5P –> 9 ADP + FUM + 15 H + HIS-L + NADH + 11 PI + SER-L + THF
	3PG + 6 ATP + 3 H2O + MLTHF + 3 NAD + 2 NADPH + 4 NH4 + OAA + R5P –> 6 ADP + FUM + 12 H + HIS-L + 3 NADH + 2 NADP + 8 PI + SER-L + THF
**L-ISOLEUCINE**	15:2:2/12:1:1
	2 ATP + CO2 + 2 H + 4 NADPH + NH4 + OAA + 2 H_M + NADPH_M + PYR_M -> 2 ADP + HCO3 + ILE-L + 4 NADP + 2 PI + CO2_M + H2O_M + NADP_M
	2 ATP + CO2 + 2 H + NADH + 3 NADPH + NH4 + OAA + 2 H_M + NADPH_M + PYR_M -> 2 ADP + HCO3 + ILE-L + NAD + 3 NADP + 2 PI + CO2_M + H2O_M + NADP_M
**L-LEUCINE**	11:1:1/10:1:1
	H + NAD + NADPH + NH4 + ACCOA_M + H_M + NADPH_M + 2 PYR_M ->
	CO2 + H2O + LEU-L + NADH + NADP + CO2_M + COA_M + NADP_M
**L-LYSINE**	13:2:2/11:1:1
	AMP + 3 ATP + NAD + 4 NADPH + 2 NH4 + PPI + ACCOA_M + AKG_M + H2O_M + NAD_M -> 4 ADP + LYS-L + NADH + 4 NADP + 4 PI + CO2_M + COA_M + H_M + NADH_M AMP + 3 ATP + 3 NADPH + 2 NH4 + PPI + ACCOA_M + AKG_M + H2O_M + NAD_M -> 4 ADP + LYS-L + 3 NADP + 4 PI + CO2_M + COA_M + H_M + NADH_M
**L-METHIONINE**	20:4:4/25:6:2
	3PG + ACCOA + 2 AMP + 6 ATP + H + MLTHF + NAD + 9 NADPH + 2 NH4 + OAA + 2 PPI + SO4 -> AC + 8 ADP + COA + MET-L + NADH + 9 NADP + 9 PI + SER-L + THF
	3PG + ACCOA + 2 AMP + 6 ATP + H + MLTHF + 8 NADPH + 2 NH4 + OAA + 2 PPI + SO4 -> AC + 8 ADP + COA + MET-L + 8 NADP + 9 PI + SER-L + THF
	ACCOA + 2 AMP + 6 ATP + GLX + 3 H + MLTHF + 9 NADPH + NH4 + OAA + 2 PPI + SO4 + NH4_M -> AC + 8 ADP + COA + GLY + H2O + MET-L + 9 NADP + 8 PI + THF
	ACCOA + 2 AMP + 6 ATP + GLX + 3 H + MLTHF + NADH + 8 NADPH + NH4 + OAA + 2 PPI + SO4 + NH4_M -> AC + 8 ADP + COA + GLY + H2O + MET-L + NAD + 8 NADP + 8 PI + THF
**L-PHENYLALANINE**	11:1:1/11:1:1
	ATP + E4P + 2 NADPH + NH4 + 2 PEP -> ADP + CO2 + 2 H2O + H + 2 NADP + PHE-L + 4 PI
**L-PROLINE**	6:2:2/5:1:1
	AKG + ATP + CO2 + H + NADH + 2 NADPH + NH4 -> ADP + H2O + HCO3 + NAD + 2 NADP + PI + PRO-L
	AKG + ATP + CO2 + H + 3 NADPH + NH4 -> ADP + H2O + HCO3 + 3 NADP + PI + PRO-L
**L-SERINE**	4:2:2/4:1:1
	3PG + NAD + NADPH + NH4 -> H + NADH + NADP + PI + SER-L* 2 GLX + 2 H + 2 NADPH + NH4 + NH4_M -> 2 GLY + 2 H2O + 2 NADP
**L-THREONINE**	8:2:2/7:1:1
	2 ATP + CO2 + H2O + H + **3 NADPH** + NH4 + OAA -> 2 ADP + HCO3 + **3 NADP** + 2 PI + THR-L
	2 ATP + CO2 + H2O + H + **NADH + 2 NADPH** + NH4 + OAA -> 2 ADP + HCO3 + **NAD** + 2 NADP + 2 PI + THR-L
**L-TRYPTOPHAN**	20:4:4/17:2:2
	3PG + 4 ATP + E4P + NAD + 2 NADPH + 2 NH4 + 2 PEP + R5P -> 4 ADP + CO2 + G3P + 7 H + H2O + NADH + 2 NADP + 8 PI + PYR + TRP-L
	3PG + 5 ATP + E4P + NADPH + 2 NH4 + 2 PEP + R5P -> 5 ADP + CO2 + G3P + 8 H + NADP + 9 PI + PYR + TRP-L 6 ATP + E4P + 2 GLX + 2 NADH + NADPH + 2 NH4 + 2 PEP + R5P + SER-L + NH4_M -> 6 ADP + CO2 + G3P + 2 GLY + 6 H + H2O + 2 NAD + NADP + 9 PI + PYR + TRP-L 4 ATP + E4P + 2 GLX + 3 NADPH + 2 NH4 + 2 PEP + R5P + SER-L + NH4_M -> 4 ADP + CO2 + G3P + 2 GLY + 4 H + 3 H2O + 3 NADP + 7 PI + PYR + TRP-L
**L-TYROSINE**	11:2:2/11:1:1
	ATP + E4P + NADPH + NH4 + 2 PEP -> ADP + CO2 + 2 H + H2O + NADP + 4 PI + TYR-L *
	ATP + E4P + NAD + 2 NADPH + NH4 + 2 PEP -> ADP + CO2 + 2 H + H2O + NADH + 2 NADP + 4 PI + TYR-L
**L-VALINE**	6:1:1/5:1:1
	H + NADPH + NH4 + 2 H_M + NADPH_M + 2 PYR_M -> H2O + NADP + VAL-L + CO2_M + H2O_M + NADP_M

The numbers (i:j:k) represent i, size of subnetworks for amino acids (2:1:1), j, the number of alternative subnetworks (2:1:1) and k, the number of unique lumped reactions (2:1:1), respectively. The first (i:j:k) belongs to *S*. *cerevisiae*, the second (i:j:k) belongs to *E*. *coli* for comparison. In this table the size corresponds only to smallest size subnetworks ($S_{min}^{j}$) for each BBB. Highlighted metabolites show the differences between alternative lumped reactions. (*) Lumped reactions are common with *E*. *coli*.

As a general comparison, lumpGEM generated 2797 subnetworks and 615 unique lumped reactions for 102 biomass building blocks for *E*. *coli*, whereas it generated 155 subnetworks and 114 lumped reactions for *S*. *cerevisiae* for 44 biomass building blocks (S1 File). Even though the numbers seems very different, the main explosion of number of possible subnetworks for *E*. *coli* emerges from lipids, which are not common with *S*. *cerevisiae*. Moreover, *E*. *coli* GEM has a more detailed biomass building block definition compared to *S*. *cerevisiae* GEM. Thus, it is fairer to compare the common biomass building blocks between two organisms as we have shown for amino acids.

We have also performed an analysis to compare *E*. *coli* and *S*. *cerevisae* for the production of a valuable industrial chemical, succinate. In this case, we did not define any core, other than the media composition, and minimized the number of active reactions to produce succinate. The analysis indicated that *S*. *cerevisiae* requires at least 33 reactions to produce succinate with a 100% carbon-mole yield over glucose, whereas *E*. *coli* needs only 25. The overall lumped reaction for *E*. *coli* do not have any metabolite other than glucose, oxygen and cytosolic proton in the reactant side, and only succinate, water and periplasmic proton on the product side. The overall lumped reaction generated for the *S*. *cerevisiae* subnetwork also includes the same metabolites, with additional production of ATP along with succinate. When we apply the same formulation to the biosynthesis of L-malate, we observe that minimal subnetwork generated for *S*. *cerevisiae* (30 reactions) are still bigger compared to *E*. *coli* (24 reactions), however this time the lumped reaction does not include any metabolite other than L-malate, glucose, water, oxygen and proton. The *E*. *coli* lumped reactions for L-malate is very similar to the succinate case. This analysis is done only for 1 *S*_*min*_ subnetwork for both compounds, and can be extended by generating all the minimum subnetworks, and for every common metabolite between *E*. *coli* and *S*. *cerevisiae*.

### Conclusion

The complexity of cellular metabolism necessitates the development of methods to better understand and investigate the metabolic capabilities of organisms. For this purpose, small sized metabolic models are developed and widely used to study cellular physiology, and are proven to be useful platforms for many applications. With the emergence of genome scale models (GEMs), studies on metabolism entered a new era, since GEMs encapsulate all biochemistry that occurs in an organism. However, it is still crucial to be able to focus on certain parts of the metabolism with a modular manner and to understand the capabilities of these modules. Within this scope, we developed lumpGEM, a systems biology tool that captures the minimal sized subnetworks that are capable of producing target compounds from a set of defined core metabolites. In this work, we applied lumpGEM on all biomass building blocks (BBB) of *E*. *coli* iJO1366 and *S*. *cerevisiae* iMM904 and generated different subnetworks that can participate in producing biomass. We also used lumpGEM to re-define the pathway and subsystem definitions for the biosynthesis of BBBs, and identified that many different subsystems participate for the production of a single BBB. Moreover, by lumping the generated subnetworks, lumpGEM allowed us to identify the individual contribution of core metabolites and cofactors for the synthesis of each BBB. This procedure is a very promising method for many applications, such as experimental studies like MFA [44] and *in silico* studies like FBA, TFA [45,46] and atom mapping analysis. The main advantage of lumpGEM is its capability of making the *ad-hoc* selected core models consistent with genome-scale model (GEMs) in terms of biomass requirements. Metabolic Flux Analysis (MFA) analysis can benefit from such approach, since lumpGEM identifies the expenditure of core metabolites for the biosynthesis of biomass building blocks, thus accounting correctly for the metabolic costs.

lumpGEM can also be used to build synthesis pathways for any metabolite defined in the metabolic network. This makes it a versatile tool to study the characteristics of industrial chemical production strains [47], since it identifies all the enzymes, either linearly connected or nested that participate in the biosynthesis of the target compound. This approach can be used to compare the similarities and differences between the host organisms to produce a target chemical, since lumpGEM can compare the metabolic costs and capabilities of different organisms for the biosynthesis of an industrially relevant molecule. Apart from pathway and subsystems/subnetworks analysis, lumping strategy reduces the complexity of the networks significantly. By exploiting this property of lumpGEM, we built a reduced model that has an *ad hoc* defined core with a biomass yield very close to its parent GEM model. With a systematic approach to define the core [48], we can generate representative reduced models that are consistent with their GEM for different studies, such as kinetic modelling [49–51], in where it is crucial to base the analysis on models that do not sacrifice stoichiometric, thermodynamic and physiological constraints.

## Materials and methods

The algorithm considers a core system of metabolites and reactions and identifies them in genome scale model (GEM) of the organism. Using this GEM, it decomposes the biomass composition of the organisms into individual biomass building blocks (BBB). lumpGEM source code is available in S3 File.

In lumpGEM, we introduce and use the following definitions:

### Preliminary definitions

*BBB*: Biomass building block
*M^core^*: Metabolites that belong to core system
*R^core^*: Reactions that belong to core system, in this specific case
*M^ncGEM^*: Metabolites that belong to GEM that do not belong to *M*^*core*^
*R^ncGEM^*: Reactions that belong to GEM that do not belong to *R*^*core*^
*S^j^*: Subnetwork (set of reactions) that synthesizes *BBB*_*j*_ other than *M*^*core*^ *R*^*core*^ and composed of *M*^*ncGEM*^ and *R*^*ncGEM*^
*M^sub^*: Metabolites that belong to *S*^*j*^
*R^sub^*: Reactions that belong to *S*^*j*^
*z_rxn_*: Binary decision variable that controls the flux through each *R*^*ncGEM*^. When decision is 0, the reaction is active

### Generating subnetworks for each BBB

a Decompose the biomass composition of GEM to each of its components, such as alanine, tyrosine, biotin, etc. In most available GEMs, such decomposition is available mainly in the biomass equation.
b Built a new GEM model by allowing the individual production of each *BBB*.
c Define *M*^*core*^ and *R*^*core*^.
d Split all the reactions in GEM in Step a. into forward *F*_*rxn*, *i*_ and backward *B*_*rxn*, *i*_ components.
e Create binary variables *z*_*rxn*, *i*_ for each $R_{i}^{ncGEM}$
f Generate a constraint for each *R*^*GEM*^ that will control the flux through these reactions as:
  $$F_{rxn,\;i}+B_{rxn,\;i}+C.z_{rxn,i}\leq C$$
  where C is the number of carbon atoms that the cell uptakes from its surrounding. If the cell can uptake multiple carbon sources, and the number of carbon atoms is not definite, an arbitrary big number can substitute for *C*.

Postulate 1: Binary control is unbiased to reaction directionality. This means that $R_{i}^{GEM}$ that is controlled by *z*_*rxn*, *i*_ can operate in both directions if the existing constraints (mass balance and thermodynamics) allow it.

g Apply thermodynamics constraints for *M*^*core*^ and *R*^*core*^ as defined in [46,52].
h Build the following MILP formulation for each *BBB*_*j*_:
  Maximize
  $$\overset{\#\;of\;R^{ncGEM}}{\underset{i}{\sum }}z_{rxn,i}$$
  such that:
  S.v=0 (1)
  $$v_{BBB,j}\geq n_{j,\;GEM}.\mu_{max}$$ (2)
  where,
  *v*_*BBB*, *j*_: The sink that is created in Step 1.a for *BBB*_*j*_ for its biosynthesis.
  μ_*max*_: Theoretical maximum specific growth rate for the given physiology in 1/hr units.
  *n*_*j*, *GEM*_: The stoichiometric coefficient for *BBB*_*j*_ in mmol/gDW unit as defined in original GEM.

Postulate 2: Any *M*^*core*^ is a potential precursor for the biosynthesis of *BBB*_*j*_.

Postulate 3: Maximizing for the sum of *z*_*rxn*, *i*_ results in the smallest subnetwork *S*^*j*^ to produce *BBB*_*j*_ from *M*^*core*^. This subnetwork is not necessarily composed of only linear pathways as reported in databases such as KEGG [53], SEED [54] or EcoCyc [40] etc. and may include branches.

Postulate 4: The flux distribution for each generated subnetwork cannot guarantee an optimum flux distribution that will specify the individual stoichiometric contribution of each *M*^*core*^ to synthesize *BBB*_*j*_ due to the degrees of freedom (DOF) that the system has.

Moreover, only the defined core reactions and metabolites of the GEM built in Step f for generating subnetworks is constraint with thermodynamics, and the generated subnetworks are constrained by only mass-balance.

To test the thermodynamic feasibility of these subnetworks, we have built the following MILP formulation for each *S*^*j*^:

a Generate a model comprised of:
  i *M*^*core*^ and *R*^*core*^
  ii *S*^*j*^
  iii *v*_*BBB*_
b Apply thermodynamic constraints on this model as described in [46,52].
c Minimize the sum of net flux in the subnetwork *S*^*j*^ [55–57].

Postulate 5: Minimizing the sum of net flux in *S*^*j*^ generates a stoichiometrically proportional flux distribution in the subnetwork *S*^*j*^. This leads to the exact stoichiometric expenditure of each *M*^*core*^ to synthesize *BBB*_*j*_.

d Lump *R*^*sub*^ with respect to the flux distribution obtained after minimization. This is collapsing the reactions into 1 overall reaction that is stoichiometrically equivalent to the flux distribution generated above.

### Generating alternative subnetworks for each BBB_j_

To identify alternative subnetworks for *BBB*_*j*_, GEM is further constrained with the following integer cuts constraint after generating each *S*^*j*^ with an iterative manner [33].

$$\overset{\#\;of\;R^{sub}}{\underset{k}{\sum }}z_{R_{k}^{sub}}>0$$

Postulate 6: Since $R_{k}^{sub}$ is active if only $z_{R_{k}^{sub}}=0$, the next solution will have at least 1 different reaction from the previous solution. Aftermath, the same procedure is applied for the newly generated *S*^*j*,2^.

## Supporting information

S1 File The subnetworks generated for *E*. *coli* and *S*. *cerevisiae* biomass building blocks. All generated subnetworks for all the biomass building blocks of *E*. *coli* iJO1366 and *S*. *cerevisiae* iMM904.
(ZIP)

S2 File The core networks generated for *E*. *coli* and *S*. *cerevisiae* GEMs. Core models for *E*. *coli* and *S*. *cerevisiae* including the defined core networks and selected lumped reactions.
(ZIP)

S3 File The source code of lumpGEM compatible with COBRA toolbox. MATLAB based code of lumpGEM that generates FBA feasible subnetworks and lumped reactions.
(ZIP)

S1 Table The statistics on the subnetworks and the lumped reactions generated for *E*. *coli* and *S*. *cerevisiae* biomass building blocks. (XLSX)

S2 Table The core metabolite and cofactor cost estimated by Neidhardt to produce biomass building blocks for *E*. *coli*. (XLSX)
