# Correction: *lumpGEM*: Systematic generation of subnetworks and elementally balanced lumped reactions for the biosynthesis of target metabolites

**DOI:** 10.1371/journal.pcbi.1005764

**Published:** 2017-09-19

**Authors:** The PLOS Computational Biology Staff

The funding statement for this article should read as follows:

“Ecole Polytechnique Fédérale de Lausanne (EPFL), the Swiss National Science Foundation, the European Union’s Horizon 2020 research and innovation programme under grant agreement No 686070, MicroscapesX within SystemsX.ch, the Swiss Initiative for Systems Biology evaluated by the Swiss National Science Foundation, and RobustYeast within ERA net project via SystemsX.ch. The funders had no role in study design, data collection and analysis, decision to publish, or preparation of the manuscript.”
